# Risk factors and a Bayesian network model to predict ischemic stroke in patients with dilated cardiomyopathy

**DOI:** 10.3389/fnins.2022.1043922

**Published:** 2022-11-09

**Authors:** Ze-Xin Fan, Chao-Bin Wang, Li-Bo Fang, Lin Ma, Tian-Tong Niu, Ze-Yi Wang, Jian-Feng Lu, Bo-Yi Yuan, Guang-Zhi Liu

**Affiliations:** ^1^Department of Neurology, Beijing Anzhen Hospital, Capital Medical University, Beijing, China; ^2^Department of Neurology, Beijing Fangshan District Liangxiang Hospital, Beijing, China; ^3^Department of Neurology, Beijing Fuxing Hospital, Capital Medical University, Beijing, China

**Keywords:** Bayesian network, stroke, dilated cardiomyopathy, prediction model, risk factor

## Abstract

**Objective:**

This study aimed to identify risk factors and create a predictive model for ischemic stroke (IS) in patients with dilated cardiomyopathy (DCM) using the Bayesian network (BN) approach.

**Materials and methods:**

We collected clinical data of 634 patients with DCM treated at three referral management centers in Beijing between 2016 and 2021, including 127 with and 507 without IS. The patients were randomly divided into training (441 cases) and test (193 cases) sets at a ratio of 7:3. A BN model was established using the Tabu search algorithm with the training set data and verified with the test set data. The BN and logistic regression models were compared using the area under the receiver operating characteristic curve (AUC).

**Results:**

Multivariate logistic regression analysis showed that hypertension, hyperlipidemia, atrial fibrillation/flutter, estimated glomerular filtration rate (eGFR), and intracardiac thrombosis were associated with IS. The BN model found that hyperlipidemia, atrial fibrillation (AF) or atrial flutter, eGFR, and intracardiac thrombosis were closely associated with IS. Compared to the logistic regression model, the BN model for IS performed better or equally well in the training and test sets, with respective accuracies of 83.7 and 85.5%, AUC of 0.763 [95% confidence interval (CI), 0.708–0.818] and 0.822 (95% CI, 0.748–0.896), sensitivities of 20.2 and 44.2%, and specificities of 98.3 and 97.3%.

**Conclusion:**

Hypertension, hyperlipidemia, AF or atrial flutter, low eGFR, and intracardiac thrombosis were good predictors of IS in patients with DCM. The BN model was superior to the traditional logistic regression model in predicting IS in patients with DCM and is, therefore, more suitable for early IS detection and diagnosis, and could help prevent the occurrence and recurrence of IS in this patient cohort.

## Introduction

Dilated cardiomyopathy (DCM) is a myocardial disease characterized by left ventricular (LV) dilation and systolic dysfunction in the absence of coronary artery disease or abnormal loading conditions sufficient to produce LV impairment (Elliott, 2000). DCM most frequently occurs in younger adults, and its most common clinical manifestations include congestive heart failure, sudden death, arrhythmias, and thromboembolic events (Japp et al., 2016). Ischemic stroke (IS) is a catastrophic thromboembolic complication of DCM, reported in several case reports and case series (Spengos and Vemmos, 2010; Jeon et al., 2012; Kawano et al., 2014; Zhdanova et al., 2016; Li et al., 2017). Thus,early identification of IS in patients with DCM is important because it can improve clinical outcomes and reduce medical costs. So far, many prediction models have been proposed to estimate the probability of developing stroke under certain conditions [e.g., nonvalvular atrial fibrillation (AF), transient ischaemic attack (TIA)], such as the Framingham score (D’Agostino et al., 2008), ABCD (2) score (Johnston et al., 2007), and CHA2DS2-VASc score (Lip et al., 2010). Of them, the most commonly used models is the Framingham Stroke Risk Profile,which was created using Cox proportional hazards regression modeling of Framingham Study data to identify factors that were most predictive of the 10-year probability of stroke.

In general, traditional logistic regression requires independent variables that are uncorrelated with each other, but in practice, the factors affecting the occurrence of IS are not independent and may interact with each other to form a complex relationship network. Unlike logistic regression, Bayesian network (BN) can well reflect the potential relationship and relationship strength between variables by constructing directed acyclic graph and conditional probability table (Park et al., 2018). In addition, increasing evidence has confirmed successful application of BN in medical diagnosis, expert systems, statistical decision making, learning, and prediction (Agrahari et al., 2018; Zhang et al., 2019). However, an agreed set of guidelines or reports on developing predictive models for IS in DCM cohorts are currently unavailable. Hence, there is a great need for further work toward constructing highly predictive models for early IS detection and diagnosis. This study established and compared traditional logistic regression and BN predictive models for IS occurrence using known risk factors.

## Materials and methods

### Patients and data collection

We selected 634 patients with DCM treated at three referral management centers between January 2016 and August 2021, mainly because Beijing Anzhen Hospital is one of the largest national centers for cardiovascular disease. The following inclusion criteria were used: (i) age ≥ 18 years; (ii) diagnosis of DCM following the European Society of Cardiology proposal which is based on systolic dysfunction and LV dilatation confirmed by echocardiography or cardiac magnetic resonance imaging and after excluding abnormal loading conditions or coronary artery disease (Pinto et al., 2016). The exclusion criteria were as follows: (i) patients with ischemic cardiomyopathy, rheumatic heart disease, arrhythmogenic cardiomyopathy, congenital heart disease, pulmonary heart disease, drug-induced cardiomyopathy, hypertensive heart disease, perinatal cardiomyopathy, valvular heart disease, and alcoholic cardiomyopathy; (ii) patients with missing clinical data. IS was diagnosed based on medical history, clinical examination, and cranial magnetic resonance imaging and magnetic resonance angiography scan results and confirmed by two attending neurologists.

Data collected at the first hospital admission, including demographic information, medical history, comorbidities, echocardiography, electrocardiogram, and laboratory tests, were collected from the electronic medical records. For patients with multiple admissions due to recurrent stroke, the data of the first admission were used in this study. This study followed the principles of the Declaration of Helsinki.

As Harrell (2015) stated, when developing a prediction model for dichotomous outcomes, the sample size should be at least 10 times the independent variable. In our research, 9 independent variables were finally included in multivariate analysis, and then the number of samples in each group should be at least 90. In fact, the number of cases of DCM with IS or without IS was 127 and 507, respectively, thus the sample size was enough to develop the prediction model.

### Quality control

The data extraction process from the medical records was standardized, and the investigators familiarized themselves with it before starting data retrieval for this study. Data entry followed a double-entry method. If discrepancies were found during the review process, the medical records were consulted, and the data were corrected.

### Data processing for predictive variables

Before building the predictive model, the collected data are preprocessed based on previous literatures. According to the studies by Li (Li et al., 2017) and Sharma (Sharma et al., 2000), AF and intracardiac thrombus are common risk factors for IS, as well-known risk factor for embolic complications (Orenes-Piñero et al., 2017). Hence, in this study, AF and intracardiac thrombus is used as risk factors for IS. Apart from these two variables, Deng (Deng et al., 2019) and Fukui (Fukui et al., 2017) also reported that lower estimated glomerular filtration rate (eGFR) was related to IS risk, with their predictive validity being well-verified. Thus, five basic characteristics (sex, age, AF, intracardiac thrombus and eGFR) of participants are ascertained. Additionally, according to biostatistics literature (Rosner, 2016), data will lose its measure of confidence if its missing value ratio > 30%. Therefore, for our study, some instances were removed from the dataset if they had more than 6 missing attributes (6 of 18). These missing attributes normally result from time conflicts and failures in the tests. Finally, a total of 26 instances were utilized as the primary dataset.

Logistic regression was utilized to screen for possible IS-related factors and evaluate assess their associated risk intensities. Logistic regression models were then applied to predict the IS, splitting the data into training and testing sets at a ratio of 7:3 using the random number table method. The training dataset was used to fit the prediction model (to “train” the algorithm), and then the model was utilized to predict the variable of interest from the test dataset. Similarly, a BN model of the IS-related risk factors in patients with DCM was established by a Tabu search algorithm using the training dataset. The test dataset was used to assess the models’ accuracy. Before establishing the BN model, all IS-related factors were quantified and coded (Supplementary Table 1 in Supplementary material 1).

### Bayesian networks

As a probabilistic graphical model, the BN uses directed acyclic graphs to describe the probabilistic relationships between variables (Liao et al., 2017). The directed acyclic graph nodes stand for random variables *U* = {X_*i*_, …, X_*n*_}, and the directed edges (E) stand for the probabilistic dependency relations between the variables. If a directional arc from X_1_ to X_2_ is seen, we can infer that X_1_ causes X_2_; thus, X_1_ and X_2_ are usually defined as the parent and child, respectively. Each node has a conditional probability distribution table representing the parent node’s state. The BN is a representation of the joint probability distributions of random variables *X* = {X_1_, …, X_*n*_}; thus, a probability expression can be obtained:


$$\begin{array}{l} P(X_{1},\ldots ,X_{n})=P(X_{1})P(X_{2}|X_{1})\ldots P(X_{n}|X_{1},X_{2},\ldots X_{n-1})\\ \;\;\;\;\;\;\;=\prod_{1}^{n}P(X_{i}|\pi (X_{i}) \end{array}$$

where π(X_*i*_) represents the collection of the parents of X_*i*_; π(X_*i*_) ⊆ {X_1_ …, X _*i*–1_} (Zhang et al., 2019).

In the present study, the collected dataset was utilized to construct a BN model for predicting the occurrence of IS. We extracted from the patient data 26 random variables for each instance. We initially filtered the nodes using logistic regression, in order to avoid including too many nodes and adding excessive complexity to the network structure. We then established the optimal model on the basis of Tabu search algorithm (Zhang et al., 2019).

### Statistical analysis

Statistical analysis was performed using IBM SPSS Statistics for Windows, Version 23.0 (IBM Corp., Armonk, NY, USA). Continuous variables are presented as mean ± standard deviation or median (interquartile range). Categorical variables are expressed as numbers and percentages. Normally distributed data were analyzed using the Student’s *t*-test (hematocrit, hemoglobin), and non-normally distributed data were analyzed using the Mann-Whitney *U* test [age, systolic blood pressure, leukocyte, platelet, eGFR, serum sodium (Na+), high-sensitivity C-reactive protein (Hs-CRP), D-dimer, left ventricular end-diastolic diameter, left ventricular ejection fraction, left atrium diameter, pulmonary arterial pressure]. Categorical variables were analyzed using the chi-squared test (male, smoking, drinking, hyperuricemia, hypertension, hyperlipidemia, diabetes, AF or atrial flutter, cardiac function, left bundle branch block, mitral regurgitation, and intracardiac thrombosis). Binary logistic regression analysis assessed the variables associated with DCM-related IS. Variables demonstrating an association with the outcome at a level of < 0.05 in univariate analysis were candidates for further multivariate analysis. Receiver operating characteristic analysis assessed the predictive models, and their areas under the curve (AUCs) were calculated. Furthermore, Delong test was applied to test the statistical significance of the difference between the AUC values. Hosmer–Lemeshow test and calibration plots were used to assess the calibration of each model. Statistical significance was set at *P* < 0.05. RStudio software, Version 4.2.0,^1^ was employed for structural learning of the BN and parameter estimation using the maximum likelihood estimation method. The BNs’ topology and conditional probability distribution tables were drawn using the Netica32 software (Norsys Software Corp., Vancouver, BC, Canada).

## Results

### Patients selection

**FIGURE 1:**
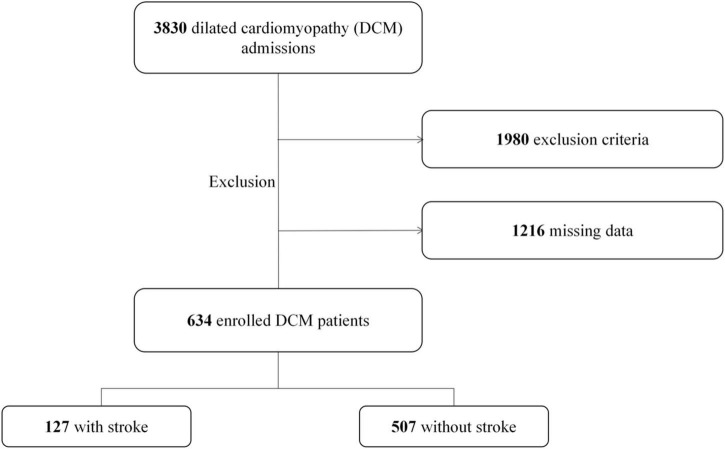
Flowchart describing the screening of patients with dilated cardiomyopathy (DCM).

Among the 3,830 patients diagnosed with DCM, 3,196 were excluded because of secondary cardiomyopathy etiologies or missing data. Finally, 634 eligible cases, including 127 with and 507 without IS were included in the study (Figure 1).

### Risk factors for ischemic stroke

Multiple variables, including basic characteristics, stroke risk factors, echocardiography findings [i.e., left ventricular end-diastolic diameter, LV ejection fraction (LVEF), and left atrium diameter], electrocardiogram, and laboratory results, were compared between patients with and without IS (Table 1). Of the 26 variables, nine were associated with IS by univariate logistic regression: hypertension [odds ratio (OR), 1.561; 95% confidence interval (CI), 1.068–2.282; *P* = 0.022], hyperlipidemia (OR, 1.548; 95% CI, 1.018–2.354; *P* = 0.041), AF or atrial flutter (OR, 1.754; 95% CI, 1.159–2.655; *P* = 0.008), eGFR (OR, 0.980; 95% CI, 0.971–0.988; *P* < 0.001), serum sodium (OR, 0.915; 95% CI, 0.865–0.968; *P* = 0.002), Hs-CRP (OR, 1.029; 95% CI, 1.010–1.048; *P* = 0.002), D-dimer (OR, 1.000; 95% CI, 1.000–1.001; *P* = 0.015), cardiac function (classes III and IV; OR, 1.720; 95% CI, 1.093–2.706; *P* = 0.019), and intracardiac thrombosis (OR, 5.682; 95% CI, 3.130–10.315; *P* < 0.001).

**TABLE 1:** Baseline data of patients with dilated cardiomyopathy (DCM).

Variables	DCM with IS (*n* = 127)	DCM without IS (*n* = 507)	*P*-value
Age, years	58 (49, 63)	56 (47, 65)	0.39
Male	96 (75.6%)	377 (74.4%)	0.776
Current smoking	38 (29.9%)	111 (21.9%)	0.056
Current drinking	30 (23.6%)	87 (17.2%)	0.093
Hyperuricemia	30 (23.6%)	87 (17.2%)	0.093
Hypertension	58 (45.7%)	169 (33.3%)	0.01
Hyperlipidemia	43 (33.9%)	126 (24.9%)	0.04
Diabetes	37 (29.1%)	122 (24.1%)	0.238
AF or atrial flutter	46 (36.2%)	125 (24.7%)	0.009
Cardiac function (class III, IV)	98 (77.2%)	336 (66.3%)	0.018
Left bundle branch block	22 (17.3%)	94 (18.5%)	0.751
Mitral regurgitation (moderate to severe)	68 (53.5%)	297 (58.6%)	0.304
Systolic blood pressure (mmHg)	116 (103, 130)	116 (102, 126)	0.372
Leukocyte (10^9^/L)	6.7 (6.0, 8.4)	6.8 (5.8, 8.1)	0.551
Hematocrit (%)	41.6 ± 5.5	42.2 ± 5.3	0.552
Platelets (10^9^/L)	198 (169, 242)	202 (169, 246)	0.45
Hemoglobin (g/L)	141.5 ± 20.9	144.6 ± 19.3	0.4
eGFR (mL/min/1.73 m^2^)	81.1 (59.4, 96.5)	90.2 (72.5, 102.1)	< 0.001
Serum Na + (mmol/L)	138.8 (136.4, 140.9)	139.7 (137.8, 141.2)	0.006
Hs-CRP (mg/L)	3.33 (0.97, 10.19)	1.82 (0.8, 5.8)	0.001
D-dimer (ng/mL)	240 (100, 611)	135 (78, 298)	< 0.001
**Echocardiography**			
LVEDD	64 (59, 71)	66 (60, 74)	0.064
LVEF	30 (25, 37)	30 (25, 37)	0.769
LAD	45 (40, 50)	45 (40, 50)	0.964
PAD	30 (25,45)	30 (25, 45)	0.45
Intracardiac thrombosis	27 (21.2%)	23 (4.5%)	< 0.001

AF, atrial fibrillation; eGFR, estimated glomerular filtration rate; Hs-CRP, high-sensitivity C-reactive protein; IS, ischemic stroke; LVEDD, left ventricular end-diastolic diameter; LVEF, left ventricular ejection fraction; LAD, left atrium diameter; PAD, pulmonary arterial pressure.

The following five significant variables were retained in the final multivariate logistic regression model after performing a backward stepwise variable selection: hypertension (OR, 1.531; 95% CI, 1.004–2.334; *P* = 0.048), hyperlipidemia (OR, 1.723; 95% CI, 1.088–2.729; *P* = 0.020), atrial fibrillation/flutter (OR, 1.597; 95% CI, 1.017–2.507; *P* = 0.042), eGFR (OR, 0.986; 95% CI, 0.977–0.995; P = 0.003), and intracardiac thrombosis (OR, 5.417; 95% CI, 2.849–10.300; *P* < 0.001; Table 2).

**TABLE 2:** Risk factors of ischemic stroke in patients with dilated cardiomyopathy (DCM): Univariate and multivariate binary logistic regression analysis.

Characteristics	Univariate analysis		Multivariate analysis	
		
	OR (95% CI)	*P*-value	OR (95% CI)	*P*-value
Hyperlipidemia	1.548 (1.018–2.354)	0.041	1.723 (1.088–2.729)	0.020
Hypertension	1.561 (1.068–2.282)	0.022	1.531 (1.004–2.334)	0.048
AF or atrial flutter	1.754 (1.159–2.655)	0.008	1.597 (1.017–2.507)	0.042
eGFR (mL/min/1.73 m^2^)	0.980 (0.971–0.988)	< 0.001	0.986 (0.977–0.995)	0.003
Serum sodium [Na](mmol/L)	0.915 (0.865–0.968)	0.002	0.965 (0.905–1.028)	0.267
Hs-CRP (mg/L)	1.029 (1.010–1.048)	0.002	1.014 (0.999–1.030)	0.071
D-dimer (ng/mL)	1.000 (1.000–1.001)	0.015	1.000 (1.0–1.0)	0.249
Cardiac function (class III, IV)	1.720 (1.093–2.706)	0.019	1.205 (0.732–1.981)	0.463
Intracardiac thrombosis	5.682 (3.130–10.315)	< 0.001	5.417 (2.849–10.300)	< 0.001

AF, atrial fibrillation; eGFR, estimated glomerular filtration rate; Hs-CRP, high-sensitivity C-reactive protein.

### Bayesian network structure

**FIGURE 2:**
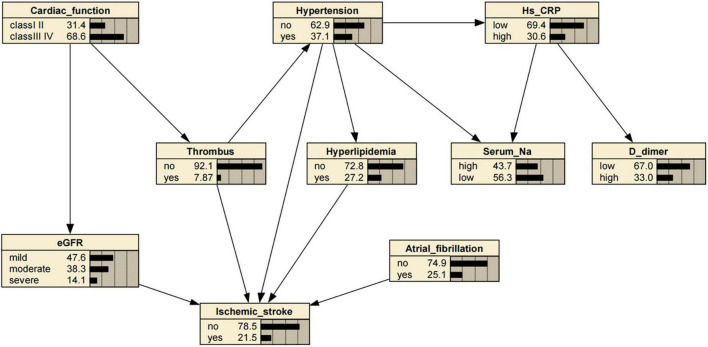
Bayesian network (BN) for predicting occurrence of ischemic stroke (IS) in patients with dilated cardiomyopathy (DCM). The BN model used nine variables selected by univariate logistic regression analysis. Estimated glomerular filtration rate (eGFR), high-sensitivity C-reactive protein (Hs–CRP), Serum sodium [Na], and D-dimer levels were defined according to their values. eGFR ml/min/1.73 m^2^: mild (≥ 90), moderate (60–90), severe (≤ 60). Hs-CRP levels (mg/L): low (< 5), high (≥ 5). Serum sodium [Na] levels (mmol/L): high (≥ 140), low (< 140). D-dimer levels (ng/ml): low (< 240), high (≥ 240).

The BN model of the IS-related factors consisted of 10 nodes and 13 directed edges. The nodes represented IS, hypertension, hyperlipidemia, AF/atrial flutter, eGFR, serum sodium, high-sensitivity C-reactive protein, D-dimer, cardiac function (class III or IV), and intracardiac thrombosis. Nodes directly linked to IS through complex network relationships included hyperlipidemia, atrial fibrillation/flutter, eGFR, and intracardiac thrombosis; heart failure (cardiac function classes III and IV) was indirectly associated with eGFR and intracardiac thrombosis, and hypertension was either directly or indirectly linked with IS through its association with hyperlipidemia (Figure 2). Based on the maximum likelihood estimation, the common variables predicting IS were hypertension, hyperlipidemia, atrial fibrillation/flutter, eGFR, and intracardiac thrombosis (Table 3).

**TABLE 3:** The conditional probability table of the training set basing on ischemic stroke (IS) as the target node.

eGFR (mL/min/1.73 m^2^)	Hypertension	Hyperlipidemia	Intracardiac thrombosis	AF/atrial flutter	Ischemic stroke	
					
					Yes	No
≥ 90	yes	no	no	no	0.14	0.86
≥ 90	yes	yes	no	no	0.18	0.82
≥ 90	yes	yes	no	yes	0.29	0.71
60–90	yes	yes	yes	no	1.00	0.00
60–90	no	yes	yes	no	1.00	0.00
≥ 90	no	no	no	no	0.15	0.85
≥ 90	no	yes	no	no	0.04	0.96
≥ 90	no	no	no	yes	0.19	0.81
≥ 90	yes	no	no	yes	0.09	0.91
≥ 90	no	yes	no	yes	0.20	0.80
60–90	no	no	no	no	0.02	0.98
60–90	yes	no	no	no	0.33	0.67
60–90	yes	yes	no	no	0.22	0.78
60–90	no	yes	no	no	0.10	0.90
60–90	no	no	no	yes	0.05	0.95
60–90	yes	no	no	yes	0.14	0.86
60–90	yes	yes	no	yes	0.25	0.75
60–90	no	yes	no	yes	0.40	0.60
≤ 60	no	no	no	no	0.29	0.71
≤ 60	yes	no	no	no	0.14	0.86
≤ 60	no	yes	no	no	0.50	0.50
≤ 60	yes	yes	no	no	0.50	0.50
≤ 60	yes	no	no	yes	0.50	0.50
≤ 60	no	no	no	yes	0.12	0.88
≤ 60	no	yes	no	yes	0.50	0.50
≤ 60	yes	yes	no	yes	0.50	0.50
≥ 90	no	no	yes	no	0.38	0.62
≥ 90	no	yes	yes	no	0.00	1.00
≥ 90	yes	no	yes	yes	0.00	1.00
≥ 90	yes	yes	yes	yes	0.00	1.00
60–90	no	no	yes	no	0.20	0.80
60–90	no	no	yes	yes	0.67	0.33
60–90	yes	no	yes	yes	0.00	1.00
60–90	no	yes	yes	yes	1.00	0.00
≤ 60	no	no	yes	no	0.75	0.25
≤ 60	no	no	yes	yes	1.00	0.00

### Model performance evaluation

**FIGURE 3:**
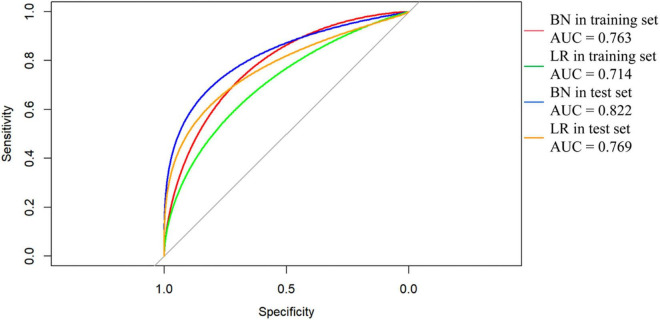
Receiver operating characteristic (ROC) curves of Bayesian network (BN) model and logistic regression (LR) model for predicting ischemic stroke (IS) in patients with dilated cardiomyopathy (DCM). The areas under the curve (AUC) of BN model predicting IS was 0.763 (95% CI, 0.708–0.818) and 0.822 (95% CI, 0.748–0.896) in (red line) training and (blue line) test datasets, respectively. The AUC of LR model predicting IS was 0.714 (95% CI, 0.649–0.778) and 0.769 (95% CI, 0.674–0.864) in (green line) training and (orange line) test datasets.

**FIGURE 4:**
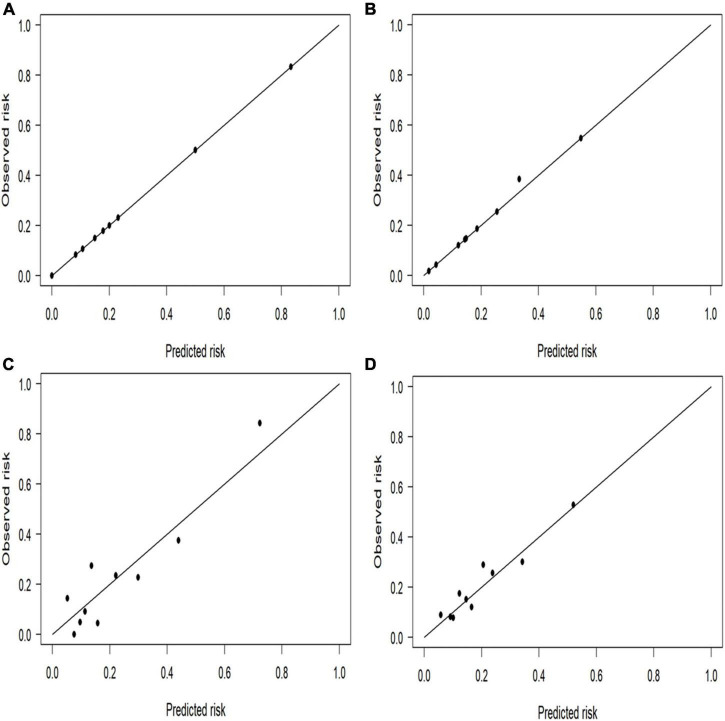
Calibration plots for the four prediction models in both cohorts. The perfect prediction should be on the 45-degree line. The calibration plots showed that the predicted risk of ischemic stroke (IS) agreed well with the observed risk, in either Bayesian network model of **(A)** test and **(B)** training datasets, or in logistic regression model of **(C)** test and **(D)** training datasets.

Compared with the logistic regression predictive model, the BN model for predicting IS achieved higher or equal scores in the training and test datasets (Table 4). The BN model achieved accuracies of 83.7 and 85.5%, AUCs of 0.763 (95% CI, 0.708–0.818) and 0.822 (95% CI, 0.748–0.896), sensitivities of 20.2 and 44.2%, and specificities of 98.3 and 97.3% in the training and test datasets, respectively. The logistic regression predictive model achieved accuracies of 83.0 and 84.5%, AUCs of 0.714 (95% CI, 0.649–0.778) and 0.769 (95% CI, 0.674–0.864), sensitivities of 17.9 and 39.5%, and the same specificities as the BN model (Figure 3). However, the Delong test revealed that there were no statistical differences in the AUC values between BN model and logistic regression model in either training datasets or test cohorts (*P* = 0.199 or *P* = 0.388). In addition, the calibration plots showed that the predicted probabilities of IS agreed well with the actual observations (Figure 4), and the Hosmer–Lemeshow test also demonstrated good calibration for BN model in training sets (*P* = 0.9999, chi square = 0.462, degree of freedom = 8) and test sets (*P* > 0.9999, chi square = 0, degree of freedom = 8), as well as for logistic regression model in training sets (*P* = 0.8234, chi square = 4.359, degree of freedom = 8) and test sets (*P* = 0.1028, chi square = 13.273, degree of freedom = 8).

**TABLE 4:** The performance of different predictive models.

Model	Accuracy	AUC	Sensitivity	Specificity
Bayesian network (training set)	83.67%	0.763	20.23%	98.32%
Logistic regression (training set)	82.99%	0.714	17.86%	98.32%
Bayesian network (test set)	85.49%	0.822	44.19%	97.33%
Logistic regression (test set)	84.45%	0.769	39.53%	97.33%

AUC, area under the curve.

## Discussion

Generally, disease risk prediction requires a statistical risk factor model (Zhang et al., 2016). The present study used univariate and multivariate logistic regression models to screen the main risk factors for IS in patients with DCM. Subsequently, we constructed a BN model to estimate the conditional probability of each node based on the univariate analysis using the Tabu search algorithm. Our BN analysis suggested that hypertension, hyperlipidemia, AF or atrial flutter, eGFR, and intracardiac thrombosis was directly associated with IS, while cardiac insufficiency (i.e., heart failure) was indirectly linked to IS through eGFR and intracardiac thrombosis. Our findings are consistent with a retrospective case series of cardioembolic strokes with hypertrophic cardiomyopathy (*n* = 8) or DCM (*n* = 12), showing that over half of the patients with DCM had reduced LVEF (< 40%), enlarged left ventricular end-diastolic dimension (> 5.6 cm) and left atrium diameter (> 4 cm), and most (60%) had documented sinus rhythm when AF was diagnosed at stroke onset or during follow-up (Li et al., 2017). Together with well-known cardiovascular risk factors, such as hypertension and hyperlipidemia (O’Donnell et al., 2010; Wang et al., 2022), these risk factors could prompt or contribute to the formation of intracardiac thrombi, resulting in cardioembolic stroke (Crawford et al., 2004; Li et al., 2017). Moreover, a retrospective cohort study by Deng et al. reported that decreased eGFR (≤ 60 mL/min/1.73 m^2^) was associated with IS in patients with DCM (Deng et al., 2019). However, the underlying mechanism remains uncertain; therefore, we can only speculate that decreased eGFR in patients with DCM promotes the formation of thrombi through excessive oxidative stress on the vascular endothelium and activation of the renin-angiotensin system. Nonetheless, more evidence is required to address these issues.

In our study, cardiac insufficiency (i.e., heart failure) was indirectly linked to IS through eGFR and intracardiac thrombosis. This is noteworthy as a study by Kostas et al. revealed that heart failure, as a predictor independent of age, sex, stroke severity, and other stroke-related risk factors, could predict death in patients with stroke (Vemmos et al., 2012). Under pathophysiological conditions, patients with heart failure often have a decreased LVEF and abnormal intracardiac blood flow due to LV systolic dysfunction caused by LV dilation. Furthermore, endothelial dysfunction and changes in blood components (e.g., platelet function) have been observed in some patients with heart failure but normal LVEF, contributing to increased susceptibility to thromboembolism (Schumacher et al., 2018). Heart failure development might activate the sympathetic nervous system and the renin-angiotensin-aldosterone system, leading to constriction of glomerular afferent arterioles and decreased glomerular filtration rate and renal blood flow due to low cardiac output (Braunwald, 2019). Therefore, further investigation should determine the role of heart failure in the pathogenesis of IS in patients with DCM and whether timely therapy to improve cardiac function could reduce the occurrence of IS.

Bayesian network (BN) models possess certain advantages in the medical domain, including adaptability and strong robustness against missing values (Sheng et al., 2019). As to adaptability, building the BN model can start with limited domain knowledge, which is then simplified or extended by inputting new knowledge to meet various needs. Clinicians can add patients’ updated knowledge, letting the BN model automatically adjust the probabilities. As to strong robustness against missing values, the BN model does not need complete knowledge of the topic; it can utilize available knowledge to perform its prediction. The BN model has been used to infer the probability of IS in patients with DCM. As shown in Table 3, patients with hypertension but without hyperlipidemia, abnormal renal function, intracardiac thrombosis, and AF or atrial flatter had a probability of 0.14 for concurrent IS; if the patient had hypertension and hyperlipidemia, the probability was 0.18; if the patients had atrial fibrillation/flatter, hypertension, and hyperlipidemia, the probability increased to 0.29; if the patient’s eGFR was 60–90 mL/min/1.73 m^2^, with hyperlipidemia, intracardiac thrombosis, but without AF or atrial flatter, the probability was 1.0. Hence, our results substantiated that the BN model based on the Tabu search algorithm had a flexible inference mechanism, making it very helpful for early IS detection and diagnosis in patients with DCM and, more importantly, for preventing the occurrence and recurrence of IS.

Besides its ability to generate an interpretable prediction and reduced uncertainty, BN is a powerful machine learning method to classify imbalanced datasets (Drummond and Holte, 2003; Monsalve-Torra et al., 2016), an important feature because a class imbalance is one of the most important challenges in real-world studies (Maldonado et al., 2014). In our study, calibration was good for both BN model and logistic regression model. Besides, the performance of our proposed BN model was promising and satisfactory in terms of accuracy, AUC, sensitivity, and specificity when compared to the traditional logistic regression model, albeit not statistically significant (e.g., AUC). This is possibly because logistic regression relies on independent variables, but the clinical features of IS and related factors are not independent; complex interaction networks might exist among them. Applied logistic regression models can predict the probability of developing IS until the state of the variables is known; however, in clinical practice, factors utilized for model prediction might be missing, leading to their inability to predict (Lee et al., 2005). In contrast the BN is constructed based on disease-related knowledge, fully mining potential information from the data and revealing the multilevel interactions between multiple factors. Additionally, the BN can outperform the radial basis function and multilayer perceptron in terms of sensitivity (Monsalve-Torra et al., 2016). In contrast, BN achieved a sensitivity of approximately 40% for identifying IS in our study. Three possible reasons for the imperfect sensitivity of our BN model were hypothesized. (i) The used dataset was not complex (contained only 26 attributes). The included attributes were derived from general information, including the subjects’ basic characteristics and simple accessory tests, rather than special radiographic data such as brain neuroimaging. The main reason for using such a dataset was to develop a predictive model for IS that can be easily utilized in community clinics or rural hospitals. Hence, special neuroimaging data that might have improved its performance could not be included. (ii) The dataset used was not large (*n* = 634). The identification accuracy would undoubtedly be increased if a larger dataset was utilized (Wang et al., 2014). (iii) Skewed dataset could impact the model’s performance (Watt and Bui, 2008); for example, males comprised 70% of the patients. Therefore, the reliability and validity of the BN model could be improved by using advanced learning algorithms.

In conclusion, our study is the first to propose a BN model to predict IS in patients with DCM, achieving a better performance than the traditional logistic regression model. Hypertension, hyperlipidemia, AF or atrial flutter, lower eGFR, and intracardiac thrombosis were good predictors of IS in our patient cohort. However, this study had some limitations. First, the number of patients with DCM complicated by IS was small. Second, as a retrospective study, clinical and laboratory data (e.g., troponin and B-type natriuretic peptide) were incomplete. Finally, the BN-directed edges reflected probability dependence between variables rather than a causal relationship. Therefore, long-term, multicenter prospective studies should be conducted to gain more insights into the potential causal relationship between the risk factors and IS in patients with DCM, optimize disease prevention strategies, and ultimately improve the long-term survival of patients with DCM.

## Data availability statement

The raw data supporting the conclusions of this article will be made available by the authors, without undue reservation.

## Ethics statement

The studies involving human participants were reviewed and approved by Research Ethical Committee of Beijing Anzhen Hospital, Beijing Fangshan District Liangxiang Hospital, and Beijing Fuxing Hospital. The patients/participants provided their written informed consent to participate in this study.

## Author contributions

G-ZL conceived the experiments. Z-XF, C-BW, and L-BF conducted the experiments. LM, T-TN, Z-YW, J-FL, and B-YY analyzed the results. All authors reviewed the manuscript.

## Footnotes

1
https://www.rstudio.com/

## Abbreviations

AUC: area under the receiver operating characteristic curve
BN: Bayesian network
CI: confidence interval
DCM: dilated cardiomyopathy
eGFR: estimated glomerular filtration rate
IS: ischemic stroke
LV: left ventricular
LVEF: left ventricular ejection fraction
OR: odds ratio.
