# Supplementary material for: Risk factors and a Bayesian network model to predict ischemic stroke in patients with dilated cardiomyopathy
Source: Front Neurosci. 2022 Nov 9;16:1043922. doi: 10.3389/fnins.2022.1043922 (PMC9683474; doi:10.3389/fnins.2022.1043922)
Supplement: Supplementary file 1 [file Table_1.docx]

**Supplementary Table 1** Risk factors and assignment of ischemic stroke (IS) in dilated cardiomyopathy (DCM)

| **Variables** | **Assignment** |
| --- | --- |
| Hypertension | no = 0, yes = 1 |
| Hyperlipidemia | no = 0, yes = 1 |
| D-dimer (ng/mL) | ≥ 240 = 1, < 240 = 0 |
| Atrial fibrillation or atrial flutter | no = 0, yes = 1 |
| Cardiac function (class Ⅲ, Ⅳ) | no = 0, yes = 1 |
| eGFR (mL/min/1.73 m^2^) | ≥ 90 = 0, 60–90 = 1, ≤ 60 = 2 |
| Intracardiac thrombosis | no = 0, yes = 1 |
| Serum Na+ (mmol/L) | ≥ 140 = 0, < 140 = 1 |
| Hs-CRP (mg/L) | ≥ 5 = 1, < 5 = 0 |
| Ischemic stroke | no = 0, yes = 1 |

eGFR, estimated glomerular filtration rate; Hs-CRP, high-sensitivity C-reactive protein
